# 

**Published:** 1883-05

**Authors:** 


					﻿MAY, 1883.
THIRTI ETH YEAR.
IIATÆS
JOURNAL
OF
HEALTH
Guaranteed Circulation 200,000 Copies in 12 Months.
E. H. GIBBS, A.M., M.D., Editor,
Ko. 21 OIINTON PLACE, EIGHTH 8TBEET, NEW YOBK.
> TERMS : $1 a Year.
SiDile number, 10 cte,<
'	fl
Entered as Second-class Matter in the Post-Office in New York.
				

